# Searching for primaries in patients with neuroendocrine tumors (NET) of unknown primary and clinically suspected NET: Evaluation of Ga-68 DOTATOC PET/CT and In-111 DTPA octreotide SPECT/CT

**DOI:** 10.2478/raon-2014-0018

**Published:** 2014-11-05

**Authors:** Nils Friedemann Schreiter, Ann-Mirja Bartels, Vera Froeling, Ingo Steffen, Ulrich-Frank Pape, Alexander Beck, Bernd Hamm, Winfried Brenner, Rainer Röttgen

**Affiliations:** 1 Department of Nuclear Medicine, Charité – Universitätsmedizin Berlin, Augustenburger Platz 1, 13353 Berlin, Germany; 2 Department of Gastroenterology, Charité – Universitätsmedizin Berlin, Augustenburger Platz 1, 13353 Berlin, Germany; 3 Department of Radiology, Charité – Universitätsmedizin Berlin, Augustenburger Platz 1, 13353 Berlin, Germany

**Keywords:** NET, CUP, Ga-68 DOTATOC PET/CT, In-111 DTPA octreotide SPECT, clinically suspected NET

## Abstract

**Background:**

To evaluate the clinical efficacy of In-111 DTPA octreotide SPECT/CT and Ga-68 DOTATOC PET/CT for detection of primary tumors in patients with either neuroendocrine tumor of unknown primary (NETUP) or clinically suspected primary NET (SNET).

**Patients and methods.:**

A total of 123 patients were included from 2006 to 2009, 52 received Ga-68 DOTATOC PET/CT (NETUP, 33; SNET, 19) and 71 underwent In-111 DTPA octreotide SPECT/CT (50; 21). The standard of reference included histopathology or clinical verification based on follow-up examinations.

**Results:**

In the NETUP group Ga-68 DOTATOC detected primaries in 15 patients (45.5%) and In-111 DTPA octreotide in 4 patients (8%) (p < 0.001); in the SNET group, only 2 primaries could be detected, all by Ga-68 DOTATOC. In patients with NETUP, primary tumors could be found significantly more often than in patients with SNET (p = 0.01). Out of these 21 patients 14 patients were operated.

**Conclusion:**

Ga-68 DOTATOC PET/CT is preferable to In-111 DTPA octreotide SPECT/CT when searching for primary NETs in patients with NETUP but should be used with caution in patients with SNET.

## Introduction

Neuroendocrine tumors (NET) are a rare heterogeneous group of tumors with an increasing incidence.^1^–^3^ Arising from the endocrine cells of the diffuse neuroendocrine system of the human body, NET can occur in different body regions.^4^,^5^ With the density of neuroendocrine cells varying between different body tissues, primary NET are most common in the gastrointestinal tract and in the bronchopulmonary system.^6^,^7^ Cases where histology suggests metastasis from NET without a known primary tumor are categorized as cancer of unknown primary (CUP). CUP patients constitute 7.6–15% of NET study populations 2,4,7–9, while NET account for less than 5% of all CUP.^10^ NET patients with an unknown primary (NETUP) have a poorer prognosis than other NET patients.^6^ Kirshborn *et al.* reported a 10-year survival rate of 22%.^6^,^8^ Surgical management is the only curative approach and should always be considered as a treatment option even when resection appears to be difficult and metastasis is present.^11^–^14^

Excellent diagnostic imaging is pivotal for optimal surgical planning. The most important imaging modalities proposed in the European neuroendocrine tumor society (ENETS) Consensus Guidelines are computed tomography (CT), magnetic resonance imaging (MRI), ultrasonography (US), contrast-enhanced US (CEUS), endoscopic US (EUS), and intraoperative US (IOUS).^15^ Because these modalities provide complementary information, most NET patients undergo diagnostic workup with a combination of imaging tests. NET cells are characterized by an increased expression of somatostatin receptors, making somatostatin receptor imaging a promising option for detecting NET.^16^–^21^ In-111 DTPA octreotide SPECT/CT is currently the standard technique for performing somatostatin receptor imaging.^22^ A promising alternative is somatostatin receptor PET/CT using tracers such as Ga-68 DOTATOC, Ga-68 DOTATATE, or Ga-68 DOTANOC. The tracer we used in this study, Ga-68 DOTATOC, was found to be more sensitive and specific than In-111 DTPA octreotide^17^, resulting in the detection of more NET lesions.^23^ Moreover, Ga-68 DOTATOC reduces patients’ radiation exposure and can be produced at low costs by specialized centers.^24^,^25^ However, as with the other PET tracers mentioned above and unlike In-111 DTPA octreotide, Ga-68 DOTATOC is not a fully approved drug in the EU and USA.

New developments in molecular NET imaging range from the combination of F-18 FDG and Ga-68 DOTATOC to characterize different tumors and their aggressiveness to promising new tracers such as Glucagon-Like-Peptide-1 (GLP-1) receptor or somatostatin receptor antagonists tracers.^26^,^27^ Depending on the clinical problem not all examinations need to have the best tumor detection or the best tumor to background ratio. If a clinician wants to be informed about the somatostatin receptor expression of a disseminated NET before planning his therapy it is not necessary to use the examination with the best lesion detection. The results of our study should help to decide which examination should be used in the search for NET primaries.

In the present study, we evaluate the performance of Ga-68 DOTATOC PET/CT and In-111 DTPA octreotide SPECT/CT in detecting unknown NET primaries. A distinction is made between patients with NETUP and patients with clinically suspected NET (SNET). Our aim is to determine whether the reported advantages of Ga-68 DOTATOC PET/CT for staging of NET will lead to a therapeutically relevant increase in the detection of NET primaries.

## Patients and methods

### Ethical adherence

The retrospective study was approved by the institutional ethics review board. Procedures followed were in accordance with the Helsinki Declaration.

### Inclusion criteria

We consecutively included all patients with NETUP as diagnosed on the basis of histology of metastasis or patients with SNET who underwent diagnostic workup with Ga-68 DOTATOC PET/CT or In-111 DTPA octreotide SPECT/CT at our department over a four-year period beginning in 2006. The clinical diagnosis of SNET was established at the ENETS Center of our hospital. Patients were randomly referred for Ga-68 DOTATOC PET/CT or In-111 DTPA SPECT/CT by the referring physicians according to availability.

### Exclusion criteria

All patients in whom the primary was already confirmed by another modality were excluded. Each patient was assigned to only one group, ensuring independent groups for statistical analysis. In patients who underwent either one of the two study modalities (SPECT/CT or PET/CT) without result and who were examined later on by the other modality, only the first examination was included in the analysis. In a second approach the group of patients who underwent both examinations was analyzed separately.

### Patient population

A total of 123 patients were included, 40 (32.5%) with SNET and 83 (67.5%) with NETUP. Table 1 summarizes the patient population. Search for the primary was performed using In-111 DTPA octreotide SPECT/CT in 71 (57.7%) patients and Ga-68 DOTATOC PET/CT in 52 (42.3%) patients. In the In-111 DTPA octreotide SPECT/CT group, 21 patients (29.6%) had SNET and 50 (70.4%) NETUP. In the Ga-68 DOTATOC PET/CT group, there were 19 patients (36.5%) with SNET and 33 (63.5%) with NETUP.

Patients in the In-111 DTPA octreotide group (42 women, 29 men) had a mean age of 58.9 years (median, 62; range, 22–81 years). Mean age in the Ga-68 DOTATOC PET/CT group (34 women; 18 men) was 55.5 years (median, 57; range, 13–83 years).

The mean interval from initial diagnosis/suspected NET to the study examination (calculated for 71 patients for whom the date of initial diagnosis was available) was 13.8 months (median, 4.5 months; range, 0–202 months) for In-111 DTPA octreotide (n=42), and 11.7 months (median, 6 months; range, 0–64 months) for Ga-68 DOTATOC PET/CT (n=29).

In the group of SNET the indication for somatostatin receptor imaging was based on the clinic of the patients. Out of 40 patients, 15 patients had flush symptoms, 17 patients diarrhea, 7 patients hypoglycemia, 3 patients hyperglycemia, 5 patients intestinal ulcerations, and 2 patients an ectopic ACTH syndrome. Six patients had a multiple endocrine neoplasia type 1 (MEN1) syndrome.

### Ga-68 DOTATOC PET/CT

PET/CT examinations were performed on a Biograph 16 scanner (Siemens AG, Germany). Ga-68 DOTATOC was prepared as described by Zhernesekov et al.^28^ The mean Ga-68 DOTATOC activity administered per patient was 112.5 MBq (median, 106 MBq; range, 66–200 MBq). The PET scan was acquired at approx. 1 hour after administration in 5 – 6 bed positions using a 168 × 168 acquisition matrix. Iterative reconstruction was performed with a scatter correction using the ordered subset expectation maximization technique (OSEM) with 5 iterations and 8 subsets. The transaxial field of view (FOV) was 585 mm and the axial FOV 162 mm. Non-contrast CT or venous phase CT was used for attenuation correction. For the triphasic CT protocol, 80–100 ml Ultravist 370 (Bayer Schering, Germany) was administered, using bolus tracking for acquisition of an arterial phase (approx. 24s delay), a portal venous phase (approx. 45s delay) for an upper abdominal scan with 16 × 0.75 mm slice thickness, and a venous phase (approx. 70s delay) for an upper abdominal scan with 16 × 1.5 mm slice thickness. PET/CT was performed with a triphasic CT protocol in 40 patients, a venous phase alone in 4 patients, and unenhanced CT in 8 patients. The CT dose parameters were: 230 effective mAs and 120 kV.

### In-111-DTPA octreotide SPECT/CT

SPECT/CT and scintigraphy examinations were performed on a Hawkeye SPECT/CT system (GE Healthcare, USA). The patients were administered 180–200 MBq In-111 DTPA octreotide, provided by an external supplier (Covidien, Petten, The Netherlands). Whole-body scintigraphic series were acquired 4 h, 24 h, and 48 h after tracer injection with a SPECT/CT acquisition of the upper abdomen after 24 h, and a repeated scan after 48 h, if needed. When whole body scintigraphy detected unclear lesions outside the upper abdomen, additional SPECT/CT images of that region were acquired. Planar whole body images were acquired with continuous table feed of 5 cm/min. SPECT imaging was performed with 360°, 60 frames (30 sec/frame), 6° angulation, 128 × 128 matrix, and a 540 × 400 mm FOV. Iterative reconstruction was performed with a scatter correction using OSEM with 2 iterations and 10 subsets. The CT scan of the SPECT/CT protocol was performed with low-dose technique (1 cm slice thickness) with 35 effective mAs and 140 kV. The low dose scan was also used for attenuation correction of SPECT.

### Analysis

All image data were analyzed by an experienced resident and a senior physician on a Centricity PACS Radiology RA 1000 Workstation (GE Healthcare, USA). The readers recorded the detection of primary NET lesions, primary NET lesions in additional sites, and sites with multiple tumors. For each primary NET lesion detected, the maximum standardized uptake value (SUVmax) was determined by placing a region of interest (ROI) in transaxial attenuation-corrected PET image. SUV was calculated according the formula:

$$\mathrm{SUV}=\frac{\left(Q1/\mathrm{Qinj}\right)}{\mathrm{BW}}$$

where Q1 is the activity within the lesion in mCi/ml, Qinj the activity injected in mCi, and BW the patient’s body weight adapted standardization value in grams. PET and CT images were analyzed separately.

Data were compiled and analyzed using Excel (Microsoft, USA) and SPSS Statistics 19 (IBM, USA).

Differences in the detection of primaries between patient groups were evaluated for statistical significance using the two-sided Fisher´s exact test. A p-value of less than 0.05 was considered statistically significant.

### Reference standard

For patients with true positive findings (including patients with primaries detected by CT only and patients with positive second examination after inconclusive first examination (n=30)) who were subsequently operated on, histopathology of surgical specimens was used as the standard of reference (available for 18 patients). In the other patients (n=12), follow-up examinations using MRI, CT, Ga-68 DOTATOC PET/CT, In-111 DTPA octreotide SPECT/CT, and other imaging modalities such as endosonography and endoscopy were used for reference. For confirmation of true positive primaries in patients without a histopathologic examination, the mean follow-up period was 21.4 months (median, 16 range, 6–52 months).

The mean follow-up period in patients with false positive primaries (n=6) was 24.3 months (median, 15.5 months; range, 8–63 months). In three patients, false positive findings in the rectum and ileum were additionally ruled out by colonoscopy.

## Results

### Comparison of detection rates

Disregarding CT only positive lesions Ga-68 DOTATOC PET/CT detected markedly more primaries than In-111 DTPA octreotide SPECT/CT: (17/52 (32.7%) versus 4/71 (5.6%); p < 0.001). In the NETUP group Ga-68 DOTATOC detected 15/33 primaries (45.5%), significantly more than In-111 DTPA with 4/50 detected primaries (8%) (p < 0.001). In the 40 patients with SNET, Ga-68 DOTATOC detected 2/19 primaries (10.5%), while In-111 DTPA octreotide SPECT detected no primary. Due to the small number of cases, no significance could be reached (p = 0.573).

The difference in the detection rate of primaries with Ga-68 DOTATOC between NETUP and SNET was significant (p = 0.01). Out of these 21 patients with true positive primary detection 14 patients were operated.

One primary tumor sites was detected by multiphasic CT only, in a patient with NETUP. Table 2 lists primary tumor sites detected by patient groups for PET and SPECT rating tumors detected by CT only as undetected.

### False positive findings

There were 2/52 (3.8%) false positive findings in Ga-68 DOTATOC PET/CT versus 4/71 (5.6%) in the In-111 DTPA octreotide SPECT/CT group (all patients with CUP). The difference was statistically not significant (p = 0.651).

### Primary tumor detection

In 21 patients a primary localisation could be detected, due to the injected radiotracer. There were three cases of multiple primary tumors; three patients were diagnosed with PET/CT only. Two patients had primary tumors at two sites, one in the pancreas and duodenum, the other in the jejunum and ileum. One patient had two primary tumors in the jejunum. An overview of primary tumor localizations is given in Table 3. All primary tumors detected had a mean SUVmax of 15.7 (median, 10.5; range, 1.1–64.6). In two patients with SPECT/CT a subsequent PET/CT could detect multiple primary tumors. One patient had a primary tumor in the ileum and an additional primary tumor in the pancreas which could be seen in the CT only.

### Metastatic sites and histologic differentiation

The distribution of metastatic sites and of histologic grades was similar for both modalities. The data are summarized in Table 4.

### Patients examined with both modalities

Seventeen patients underwent Ga-68 DOTATOC PET/CT after In-111 DTPA octreotide SPECT/CT:

- Fifteen patients had unsuccessful In-111 DTPA octreotide SPECT/CT followed by Ga-68 DOTATOC PET/CT. In these patients primary tumors were detected by PET/CT in 7 patients by PET and in one patient by CT only.
- In two patients whose primary tumors were detected by In-111 DTPA SPECT/CT, a Ga-68 DOTATOC PET/CT was performed for improved localization. In these patients a primary localization was detected by both modalities. In one of these patients Ga-68 DOTATOC PET/CT found an additional primary tumor localization.

Three patients underwent In-111 DTPA octreotide SPECT/CT after Ga-68 DOTATOC PET/CT:

- No primary tumor could be detected by both modalities in these patients.

**FIGURE 1.**
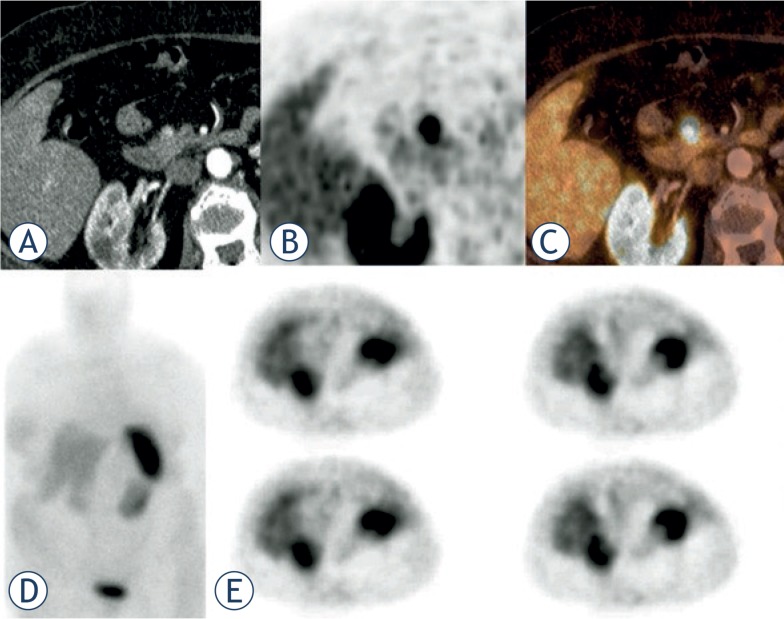
**(A–C)** Insulinoma presenting as a hyperperfused **(A)** Ga-68 DOTATOC positive lesion **(B)** in the pancreatic head **(C)**. In the In-DTPA octreotide scintigraphy **(D)** and SPECT **(E)** performed 3 days earlier no lesion could be found.

**FIGURE 2.**
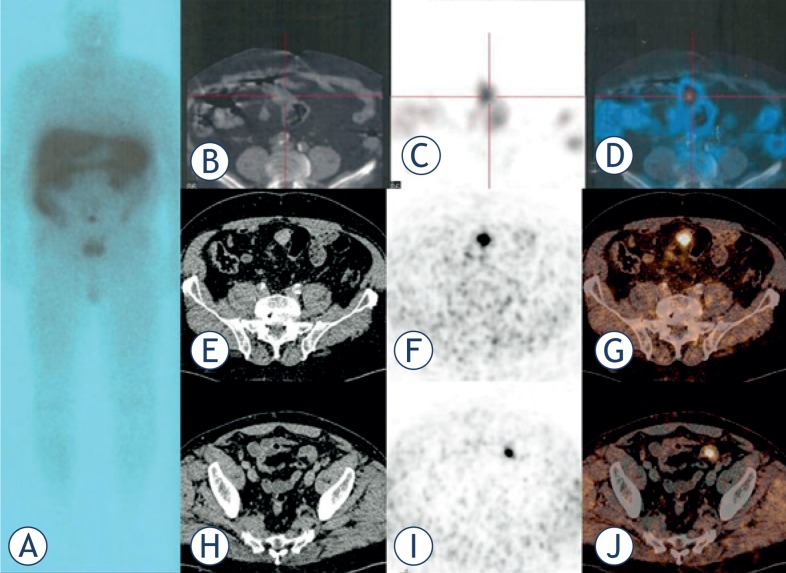
**(A)** In-111 DPTA octreotide detected one suspicious lesion located in the ileum **(B–D)** in a patient with NET liver metastases. Ga-68 DOTATOC PET/CT confirmed the lesion **(E–G)**, but could visualise also an additional lesion in the ileum undetected by In-111 DTPA octreotide **(H–J)**.

**FIGURE 3.**
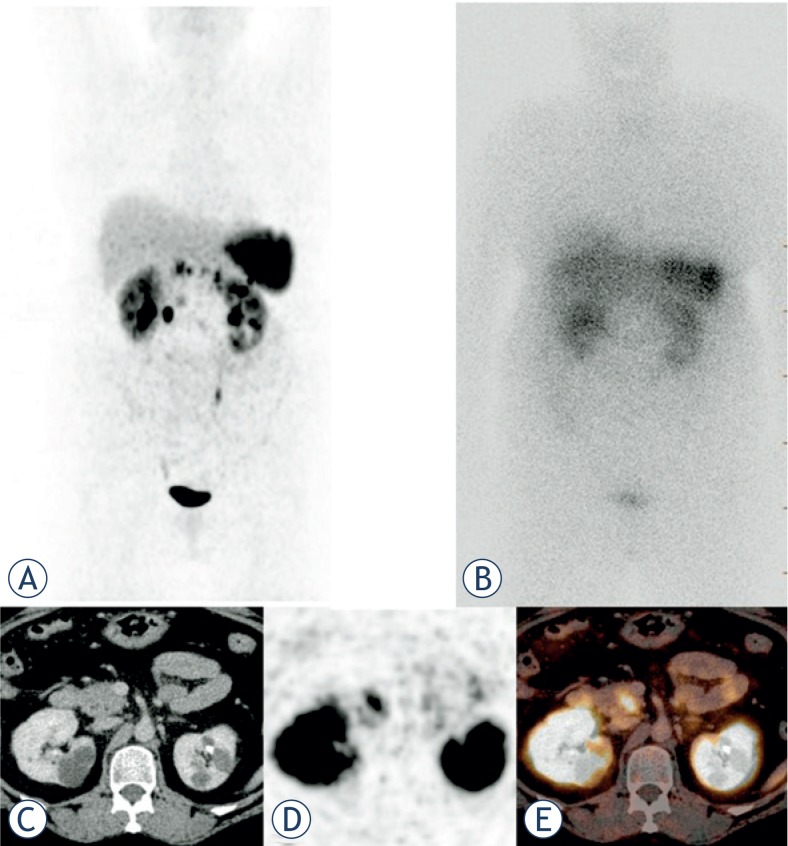
Patient with MEN1 and several NET lesions in the Ga-68 DOTATOC MIP(A), which were not visible in the In-111 octreotide scintigraphy performed a few days before (B), Ga-68 DOTATOC PET/CT images of the lesion in the pancreatic head (C–E).

Examples of patients who underwent both examinations are shown in figures 1–3.

## Discussion

Arising from cells of the diffuse neuroendocrine system, NET primaries can develop in different regions of the body.^4^ NET are rare, and only a small proportion of NET patients have cancer of unknown primary. However, the true prevalence of CUP in NET patients is likely to be higher due to documentation bias.^10^ Bias may result, for instance, when a suspected tumor is documented as a definitive diagnosis. Reported percentages must therefore be interpreted with caution. Identification of CUP by a suitable imaging modality is important because it can markedly improve patient survival. Surgery is the method of first choice in most patients with a locoregionally confined NET primary.^29^ Recent data suggest that even patients with nonresectable NET liver metastasis may benefit from resection of the primary tumor.^29^–^31^ In our study population, surgery was also a very common treatment in those patients in whom the study modalities identified a primary tumor. The sites of NETUP include the bronchi, stomach, pancreas, colon, and rectum^10^, and different imaging modalities are available to search for the primary. A clear guideline-based diagnostic strategy for identifying NETUP however does not exist. Therefore, it stands to reason to use the guidelines that exist for other CUP for orientation. The performance of different imaging modalities in identifying CUP varies with the location in which the tumor is ultimately found. For instance, EUS has excellent detection rates for tumors located in the head of pancreas^32^, while it is naturally not suitable for identifying primaries in the lungs. Somatostatin receptor imaging offers the advantage of enabling whole-body evaluation. It has gained a central role in staging NET. In-111 DTPA octreotide is the current standard for somatostatin receptor imaging^22^ and, unlike PET tracers, has been approved for marketing in the USA and Europe. The effective dose to a patient examined with In-111 DTPA octreotide SPECT/CT (12 mSv/222 MBq In-111 DTPA octreotide) is higher compared to a patient examined with Ga-68 DOTATOC PET/CT (2,5 mSV/110 MBq Ga-68 DOTATOC) including a low dose CT for attenuation correction.^24^,^25^ With superior detection rates having been reported for PET tracers^17^,^23^, we expected them to be superior to In-111 DTPA octreotide in the search for primary NET when we planned our study. However, it was difficult to estimate how much better they would perform on the basis of the available data in literature.

To the best of our knowledge, there are only two published studies that focus on somatostatin receptor imaging in NETUP.^33^,^34^ Savelli *et al*., using In-111 pentetroide scintigraphy and SPECT, reported detection of primary NET in 14/36 (39%) patients with CUP.^34^ Prasad *et al.* used Ga-68 DOTANOC, identifying 35 NET primaries in 59 patients with CUP (59%).^33^ Our detection rates for In-111 DTPA octreotide and Ga-68 DOTATOC PET/CT, 8% and 45.5%, are lower for both modalities. One important reason for this discrepancy is that populations of CUP patients are very heterogeneous with the difficulty of identifying primary tumors varying with the extent of disease, the time of first diagnosis, the number and type of prior diagnostic tests, and the clinical experience of physicians referring CUP patients. Another factor already mentioned above is how reliably and carefully the results of imaging studies are documented; this is especially important when investigating patients with SNET. We had six cases of false positive findings. The divergence of our reported detection rates is especially obvious for In-111 DTPA octreotide SPECT/CT in comparison with the study of Savelli *et al*. This we consider mainly attributable to the fact that Savelli *et al*. performed their study much earlier, *i.e*., between 1996 and 2000.^33^ Since then, there have been important technical advances, resulting in much better detection rates for MRI and CT. Hence, patients undergoing In-111 DTPA octreotide SPECT/CT today, which has not evolved much during the same period, have occult primaries after negative MR and CT imaging that are much more difficult to detect. The highest detection rate of 86.7% for occult primary tumors was reported by Wang *et al*., who focused on surgical exploration for NETUP, identifying 6/7 tumors with laparoscopy and 7/8 tumors with laparotomy in patients with well-differentiated NET liver metastases.^29^ CT and somatostatin receptor scintigraphy performed poorly with regard to the detection of primary NETUP in the gastrointestinal tract, detecting only 34.6% and 26.2%, respectively.^29^ These data were acquired between 1993 and 2008, *i.e*., predominantly later than in the study by Savelli *et al*. However, the 26.2% of primaries detected with somatostatin receptor scintigraphy also include tumors already detected by another test. Hence, the number of primary tumors first diagnosed with somatostatin receptor scintigraphy is actually lower in this study.

With Ga-68 DOTATOC and Ga-68 DOTANOC having slightly different affinity profiles for somatostatin receptor subtypes^35^, it is conceivable that the choice of tracer also may influence detection rates of NET primaries. However, this assumption remains hypothetical as we are not aware of a study comparing Ga-68 DOTATOC and Ga-68 DOTANOC in the same patient population. For comparable imaging modalities detection rates for NETUP are mainly of interest in relative terms and not absolute terms. There is good comparability of In-111 DTPA octreotide SPECT/CT and Ga-68 DOTATOC PET/CT with both being whole-body imaging modalities targeting somatostatin receptors.

In our study, Ga-68 DOTATOC PET/CT had a much better detection rate than In-111 DTPA octreotide SPECT/CT, suggesting that Ga-68 DOTATOC PET/CT should be preferred when searching for primary tumors in NETUP patients. Despite the cautionary remarks regarding the comparability of CUP patient populations made above, we think that for the purpose of our study comparability is adequate. Both imaging modalities were performed at the same center, reducing a possible bias that might result from greater heterogeneity of center specific procedures in a retrospective multicenter approach. Despite random assignment of the patients to either group, there is some indication that primary tumor detection might have been even more difficult in the Ga-68 DOTATOC PET/CT group: the Ga-68-DOTATOC PET/CT group included 15 patients with prior unsuccessful In-111 DTPA octreotide SPECT/CT, while there were only 3 patients with unsuccessful Ga-68-DOTATOC PET/CT in the In-111 DTPA octreotide SPECT/CT group. Other factors influencing the difficulty of identifying a primary NET include primary tumor localization within the body and the severity of disease as indicated by the degree of histologic differentiation and possibly the number and site of metastases. The primary tumor sites are difficult to compare between the two groups in our study due to the small number of tumors detected with In-111 DTPA octreotide SPECT/CT. The distribution of metastatic sites provides no evidence of a disadvantage for either group in this regard.

Tumor histology is an important factor influencing the detectability of lesions by somatostatin receptor imaging. This is because poorly differentiated NET have fewer somatostain receptors.^36^,^37^ Histologic grading of NETUP is still under debate.^38^ One grading system differentiates between low-grade and high-grade tumors.^10^,^38^ We used the 2010 WHO criteria, distinguishing well-differentiated low-grade (ENETS G1), intermediate grade (ENETS G2), and poorly differentiated high-grade tumors (ENETS G3).^39^ With regard to detectability based on histology, there was no advantage large enough to explain the markedly higher primary tumor detection rate of PET/CT.

The vast majority of nuclear medicine departments perform In-111 DTPA SPECT/CT without contrast medium administration for the CT scan, while most Ga-68 DOTATOC PET/CT examinations are performed with contrast administration because it has been shown to improve tumor detection.^40^,^41^ To preclude distortion, we also did a comparison of both modalities classifying only those examinations as successful in which the primary tumor was also visible on PET and rated primary tumors visible on CT only as undetected by PET/CT. This comparison was also done because the contrast administration protocols in the PET/CT examinations were not fully uniform. Such cases of lesions being detectable in the CT only would not exist under ideal conditions with patients undergoing prior CT scans within a short interval before somatostatin receptor imaging. However, it is difficult to enforce fully standardized protocols for prior examinations. The cases of primary tumors detected only by CT indicate that contrast-enhanced CT provides additional information, making lesion detection more reliable.

Ga-68 DOTATOC PET/CT also performed better in the direct comparison of those patients who underwent both imaging modalities. However, these results must be interpreted with great caution as there was usually a time interval between the two examinations. Interestingly, two of the four patients in whom the primary tumor was detected by In-111-DTPA octreotide SPECT additionally underwent Ga-68 DOTATOC PET/CT to improve lesion localization.

Enteropancreatic NET may occur in multiple locations.^7^,^42^–^45^ One possible reason is that the specific stem cells of these NET may be induced to undergo malignant transformation in different body regions by exposure to an exogenous growth factor.^7^ In a study by Katona *et al*. investigating inactivation of the X-chromosome, the majority of multilocular NET lesions of the enteropancreatic axis were found to arise independently, while some originated as a single clone with subsequent local and discontinuous metastases.^43^ In our study, some patients in whom a primary was detected had multilocular lesions. Hence, we must reconsider our concept of a single primary tumor giving rise to metastatic disease. The term CUP does not fully apply to cases of multiple NET lesions arising independently. This terminological inaccuracy must be born in mind when interpreting the results of CUP studies. An optimal imaging modality for initiating adequate therapeutic management is especially important when multilocular NET lesions are present. In this respect, somatostatin receptor PET/CT is more suitable than In-111 DTPA octreotide SPECT/CT due to its higher sensitivity as suggested by our results (multilocular NET lesions were only detected with PET/CT).

The detection rate for primary tumors was much lower in patients with SNET compared to patients with NETUP. A clinical diagnosis without histologic confirmation is highly examiner dependent. Recent studies suggest that only approx. 20% of all NET patients have typical symptoms such as Zollinger-Ellison or carcinoid syndrome.^12^,^46^,^47^ The majority of all NET patients have nonspecific symptoms.^46^,^48^–^51^ It is therefore likely that not all patients with SNET actually had NET. This is why strict criteria for ordering somatostatin receptor imaging must be used in this subset. Ga-68 DOTATOC PET/CT should be preferred in this subgroup, one reason being the lower radiation exposure. Somatostatin receptor imaging appears to be useful in patients with MEN 1 and the respective clinical presentation, as suggested by successful detection of primary tumors in three patients with MEN 1 in our study.

Our study is limited by the retrospective design, which is less accurate than a prospective study with regard to the data obtained on the number of prior examinations, date of first diagnosis, or histologic grade. A prospective study design also allows stricter randomization and better standardization of imaging procedures. An advantage of the retrospective design is the inclusion of a relatively large number of patients with this rare tumor entity. In a prospective setting, this would require a long study period for recruitment, during which tracers may become outdated.

A further limitation is that both examinations were not performed in the same collective of patients for reasons of radiation protection. However, the collectives of patients were quite comparable and there were no patient characteristics strongly favoring the Ga-68 DOTATOC patient group.

Another constriction is that histology was not available for all patients, which commonly limits other studies^33^,^34^ and was due to the fact that it is ethically precluded to operate on patients without an apparent clinical benefit for the sole reason of obtaining biopsy material. The large number of patients operated on, because the primary tumor was identified, is an advantage of our study compared to similar studies in the literature.

In conclusion, our results show that Ga-68 DOTATOC PET/CT has better detection rates compared with In-111 DTPA octreotide SPECT/CT and should be preferred to search for unknown primaries in patients with NETUP. Ideally, the protocol should include a contrast-enhanced CT scan to further improve performance. In patients with SNET, somatostatin receptor imaging should be used with caution. Its use, again preferably Ga-68 DOTATOC PET/CT, is justified only after a meticulous clinical examination which strongly suggests NET as reason for the underlying symptoms.

## Figures and Tables

**TABLE 1.** Patient population: number of patients in each group; men/women; age (mean, median, range)

	**Ga-68 DOTATOC**	**In-111-DTPA**	**All patients (n=123)**
**All patients (n = 123)**	52 (m,18;w,34)	71 (m,29;w,42)	123 (m,47;w,76)
	age: 55.5;57;13–83	age: 58.9;62;22–81	age: 57.5;59;13–83
**NETUP (n = 83)**	33 (m,13;w,20)	50 (m,24;w,26)	83 (m,46;w,37)
	age: 56.3;56;32–83	age: 61.3;66;30–81	age: 59.3;59;30–83
**SNET (n = 40)**	19 (m,5;w,14)	21 (m,5;w,16)	40 (m,10;w,30)
	age: 54.1;64;13–77	age: 53.2;54;22–72	age: 53.7;56.5;3–77

**TABLE 2.** Numbers of true positive NET primaries by modality (In-111-DTPA octreotide SPECT and Ga-68 DOTATOC PET/CT, not including primaries detected by CT only) and by patient groups

	**Ga-68 DOTATOC**	**In-111-DTPA**	**All patients (n=123)**	**p-Value**
**All patients (n = 123)**	17/52 (32.7%)	4/71 (7.1%)	21/123 (17.1%)	<0.001
**NETUP (n = 83)**	15/33 (45.5%)	4/50 (8%)	19/83 (22.3%)	<0.001
**SNET (n = 40)**	2/19 (10.5%)	0/21	2/40 (5%)	0.573
**p-Value**	0.01	0.185	0.014	

**TABLE 3.** Primary tumor sites detected based on In-111-DTPA or Ga-68 DOTATOC imaging excluding sites detected by CT only

	**lesions (n = 24) / patients (n = 21)**
**Duodenum**	4
**Jejunum**	4
**Ileum**	8
**Pancreas**	8
**Other**	0

**TABLE 4.** Sites of metastasis and histologic grades

	**Ga-68 DOTATOC (n=33)**	**In-111-DTPA (n = 50)**	**Total (n =83)**
**Metastatic site**			
Liver	23 (69.7%)	33 (66%)	66 (79.5%)
Bones	7 (21.2%)	9 (18%)	16 (19.3%)
Lymph nodes	19 (57.6%)	23 (46%)	42 (50.6%)
Lungs	2 (6.1%)	3 (6%)	5 (6%)
Other	3 (9.1%)	8 (16%)	11 (13.3%)
**Histologic grade**			
Grade 1	16 (48.5%)	21 (42%)	37 (44,6%)
Grade 2	5 (15.2%)	4 (8%)	9 (10.8%)
Grade 3	6 (18.2%)	8 (16%)	14 (16.9%)
Unknown	6 (18.2%)	17 (34%)	23 (27.7%)
